# Role of Seminal Exosomes in Reproduction

**DOI:** 10.1002/jcp.70106

**Published:** 2025-11-12

**Authors:** Shayesteh Mehdinejadiani, Nahid Azad, Zeinab Dehghan, Zahra Khosravizadeh, Fatemeh Saberi, Delsuz Rezaee, Tayyebeh Pilehchi, Nasim Goudarzi, Elnaz Salahi, Kobra Mehdinejadiani

**Affiliations:** ^1^ Faculty of Veterinary Medicine University of Calgary Calgary Alberta Canada; ^2^ Abnormal Uterine Bleeding Research Center Semnan University of Medical Sciences Semnan Iran; ^3^ Autoimmune Diseases Research Center Shiraz University of Medical Sciences Shiraz Iran; ^4^ Department of Gynecology and Obstetrics, School of Medicine Arak University of Medical Sciences Arak Iran; ^5^ Student Research Committee, Department of Medical Biotechnology, School of Advanced Technologies in Medicine Shahid Beheshti University of Medical Sciences Tehran Iran; ^6^ Department of Genetics and Molecular Medicine, School of Allied Medical Sciences Ilam University of Medical Sciences Ilam Iran; ^7^ Department of Anatomical Sciences, Cellular and Molecular Research Center, Research Institute for Prevention of Non‐Communicable Diseases Qazvin University of Medical Sciences Qazvin Iran; ^8^ Department of Obstetrics and Gynecology, Molud Infertility Center Zahedan University of Medical Sciences Zahedan Iran; ^9^ Clinical Immunology Research Center Zahedan University of Medical Sciences Zahedan Iran; ^10^ Department of Microbiology, Immunology and Infectious Diseases Calgary Univercity Calgary Alberta Canada

**Keywords:** exosomes, fertility, reproduction, seminal fluid, spermatozoa

## Abstract

Exosomes are small lipid bilayer vesicles, ranging from 30 to 150 nm in diameter, that are secreted by various cells and facilitate intercellular communication. They originate from the endosomal system and release their contents into the extracellular environment. These nanovesicles carry bioactive molecules, including nucleic acids, lipids, and predominantly proteins, influencing target cells and contributing to cell‐to‐cell interactions. Exosomes play a crucial role in both normal physiological functions and pathological conditions, including male and female reproductive disorders. Various parts of the male reproductive tract release exosomes into seminal fluid. Seminal exosomes, especially epididymosomes and prostasomes, have been shown to influence male fertility. Furthermore, the role of seminal exosomes has been demonstrated in the female reproductive tract during implantation and pregnancy. Evidence shows that the exosomal cargo in seminal fluid differs between normal and pathological conditions, impacting the reproductive process. Consequently, exosomes are considered valuable biomarkers not only for diagnosis but also for potential therapeutic roles in abnormal conditions, particularly infertility. This review aims to explore the role of seminal exosomes in male fertility and their subsequent impact on the female reproductive tract during fertilization, preimplantation, implantation, postimplantation, and pregnancy‐associated diseases, as well as the role of exosomes during seminal infections. Additionally, it aims to highlight the significance of seminal exosomes in medical applications and emphasize the need for future studies in this area.

## 1 Introduction

Seminal fluid consists of spermatozoa and seminal plasma, the acellular liquid component rich in diverse bioactive signaling molecules (Perry et al. 2013). Seminal plasma refers to the combination of secretions originating from the testis, epididymis, ductus deferens, and accessory sex glands (Rodriguez‐Martinez et al. 2021; McGraw et al. 2015). It possesses a complex and diverse composition, containing high concentrations of bioactive molecules, such as proteins, nucleic acids, metabolites, inorganic ions (including calcium, magnesium, potassium, sodium, and zinc), as well as other small molecules (Juyena and Stelletta 2012). These molecules possess the ability to circulate freely within the seminal plasma or be encapsulated within Extracellular Vesicles (EVs), protecting inhibitory factors like proteases or nucleases present in seminal plasma (Machtinger et al. 2016; Llavanera et al. 2022). EVs, originating from different organs, are a heterogeneous group of vesicles facilitating intercellular communication and regulating various biological processes. EVs, according to their characteristics and functions, can be classified into (1) apoptotic bodies (ABs), (2) microvesicles (MVs), and (3) exosomes groups (Zhou et al. 2021). In this study, we focus on exosomes, as including all EV populations could introduce interpretive uncertainty. The limited characterization of nonexosomal vesicles would necessitate speculative assumptions, potentially obscuring mechanistic insights. By concentrating specifically on exosomes, we provide a more precise and biologically grounded synthesis. Furthermore, exosomes are characterized by a favorable biosafety profile, low immunogenicity, and a remarkable capacity to transport diverse bioactive molecules. In view of their promising therapeutic potential, regulatory agencies have begun establishing frameworks for their evaluation and clinical translation. In the United States, the Food and Drug Administration (FDA) classifies exosomes as 351 biological products, requiring comprehensive assessment of their safety, efficacy, purity, and potency before clinical approval. Currently, exosome‐based therapies remain in the investigational new drug (IND) development phase. Similarly, in the European Union, exosomes fall under the regulatory category of Advanced Therapy Medicinal Products (ATMPs) (Kalodimou and Muthu 2025).

Exosomes are extremely small membranous nanovesicles, typically measuring 30–150 nm in diameter (Jeppesen et al. 2019) and function as carriers of molecular information for intercellular communication. Additionally, they play a role in various physiological and pathological processes (Farooqi et al. 2018; Aheget et al. 2020). Exosomes are enriched with a cargo of lipids, microRNAs (miRNAs), messenger RNAs (mRNAs), proteins, and other molecules, which depend on the cell of origin (Doyle and Wang 2019; Gurunathan et al. 2019). Hence, they are known as sources of circulating biomarkers (Statello et al. 2018; Buschow et al. 2005). Various cell types, including B‐cells, T‐cells, platelets, and dendritic cells, have the capability to generate exosomes (Tao et al. 2017; Ghossoub et al. 2014; Baskaran et al. 2020). These nanovesicles can be found in several body fluids, such as amniotic fluid (AF), plasma, saliva, breast milk, urine, vaginal fluid, and semen (Zhou et al. 2011; Lässer et al. 2011; Keller et al. 2011; Gallo et al. 2012; Dimov et al. 2009). Exosomes can enter target cells through three primary mechanisms: endocytosis, membrane fusion, and receptor‐ligand mediated interactions (McKelvey et al. 2015). These pathways facilitate the interaction of exosomes with recipient cells through the ligand‐receptor pathway, membrane fusion pathway, or receptor‐mediated endocytosis. Such interactions play essential roles in various biological processes, both in normal physiological functions and in pathological conditions (Urbanelli et al. 2013).

Exosomes, depending on their cellular source, play a significant role in regulating a wide range of biological activities, including cell migration, differentiation, and intercellular communication (Gurunathan et al. 2021). These nanovesicles encompass a wide range of components, with exosomes derived from various cell types identified to contain approximately 4400 proteins, 194 lipids, 1639 mRNAs, and 764 miRNAs (Mathivanan et al. 2012). Their nanoscale size, remarkable stability, biocompatibility, permeability, low toxicity, and minimal immunogenicity render exosomes ideal candidates for drug delivery systems (Sun et al. 2010; Lai et al. 2013). Indeed, exosomes have emerged as promising new biomarkers for the diagnosis and prognosis of various diseases and conditions, including reproductive development and infertility disorders (Esfandyari et al. 2021; Kowalczyk et al. 2022). The quantity, quality, and composition of exosomes reflect valuable information about the functional state of the cells from which they originate (Truong et al. 2017; Shahin et al. 2021; Pillay et al. 2019). By analyzing exosomes, researchers and clinicians can gain insights into disease progression, treatment response, and overall cellular health. This underscores the potential of exosomes as valuable tools in understanding and managing a wide range of health conditions (Donoso‐Quezada et al. 2021; del Pozo‐Acebo et al. 2021). The components within these cargoes have the potential to initiate tissue regeneration and repair processes, underscoring exosomes as a promising avenue for the development of novel diagnostic and therapeutic interventions (Ha et al. 2016).

In male infertility, the examination of seminal exosomes can provide insights into sperm quality (Murdica, Cermisoni, et al. 2019; Murdica, Giacomini, et al. 2019), reproductive function, and potential underlying causes of infertility (Barceló et al. 2018; Ma et al. 2021). Considerable research efforts have been dedicated to elucidating the importance of seminal exosomes in germ cell development, sperm maturation, sperm function, and their regulatory role in male reproduction (Yue et al. 2022; Guo et al. 2023). Semen exosomes currently hold significant potential in male mammalian reproduction, serving as biomarker candidates for identifying patients with azoospermia, teratozoospermia, and other forms of male infertility (Candenas and Chianese 2020). Additionally, in recent years, there has been a growing focus on understanding the role of paternal exosomes in the processes beyond fertilization, including preimplantation embryo development and offspring phenotypes, leading to increased attention in the scientific community (Baskaran et al. 2020; Vickram et al. 2021; Dehghan et al. 2024). Then, we aim to provide an assessment regarding the place of seminal exosomes in male fertility and subsequently during pregnancy steps in the female reproductive tract. By deepening our understanding of seminal exosomes, researchers seek to discover new perspectives that could enhance the diagnosis and management of male infertility, potentially influencing reproductive success and offspring well‐being.

## 2 Exosomes: Biogenesis, Compositions, and Functions

### 2.1 The Biogenesis of Exosomes

EVs represent a broad category that encompasses various subtypes, notably exosomes, microvesicles, and apoptotic bodies, each distinguished by their distinct cellular origins (Tannetta et al. 2014). Apoptotic bodies range in size from 50 to 5000 nm and contain DNA, RNA, and histone proteins. During apoptosis, macrophages engulf cells carrying apoptotic bodies. Microvesicles are formed through direct outward budding of the plasma membrane, giving rise to vesicles in size from approximately 50 nm to 1 μm in diameter (El Andaloussi et al. 2013; Raposo and Stoorvogel 2013). In contrast, exosomes exhibit a smaller size compared with other EVs, typically ranging from approximately 30 to 100~150 nm in diameter (Ha et al. 2016; Kalluri and LeBleu 2020). Beyond size distinctions, exosomes also differ in terms of density, cellular origin, morphology, molecular composition, and biological functions (Hessvik and Llorente 2018).

Exosomes originating from various cell types share analogous pathways in their biosynthesis, all stemming from the process of endocytosis. Initially, endocytic vesicles are formed from the plasma membrane in a process called endocytosis. These endocytic vesicles then mature into early endosomes. In the second step, the limiting membrane of the late endosomes undergoes inward budding, resulting in the formation of intraluminal vesicles (ILVs) within the lumen. The late endosomes containing these ILVs are called multivesicular bodies (MVBs). Finally, MVBs can either fuse with lysosomes for degradation or fuse with the plasma membrane of the cell through cytoskeleton proteins, leading to the release of ILVs via exocytosis into the extracellular space and body fluids. These ILVs that are released into the extracellular environment are referred to as exosomes (Essandoh and Fan 2015; Colombo et al. 2014; Trajkovic et al. 2008). Research has established the presence of a diverse array of essential proteins for exosomes biogenesis. This developmental process can occur through either ESCRT (endosomal sorting complexes required for transport)‐dependent mechanisms or through ESCRT‐independent pathways (Colombo et al. 2013; Schmidt and Teis 2012).

In the male reproductive system, the production of exosomal biomolecules in seminal plasma, mainly Prostasomes and Epididymosomes, follows a similar biogenesis process. Prostasomes, initially characterized in 1982 as vesicles measuring 50–100 nm in diameter, are generated within prostate epithelial cells through the inward budding of endosomal membranes (Stegmayr and Ronquist 1982; Ronquist and Brody 1985). Subsequently, these Prostasomes are discharged into the prostatic ductal system by the fusion of multivesicular endosomes with the apical plasma membrane of the epithelial cell (Zijlstra and Stoorvogel 2016). Epididymosomes, first identified by Yanagimachi and colleagues in 1980 (Yanagimachi et al. 1985), have since been observed in various species including mice (Rejraji et al. 2002), rats (Fornés et al. 1995), bulls (Frenette et al. 2002), and humans (Thimon et al. 2008). These vesicles are released from the epididymal epithelium through an apocrine secretory pathway, involving the formation of sizable blebs at the apical edge of the parent cell (Hermo and Jacks 2002). Ultimately, these blebs detach from the apical membrane, disintegrate within the lumen, and release their contents into the extracellular space (Aumüller et al. 1997).

### 2.2 Compositions and Biological Functions of Exosomes

**Figure 1:**
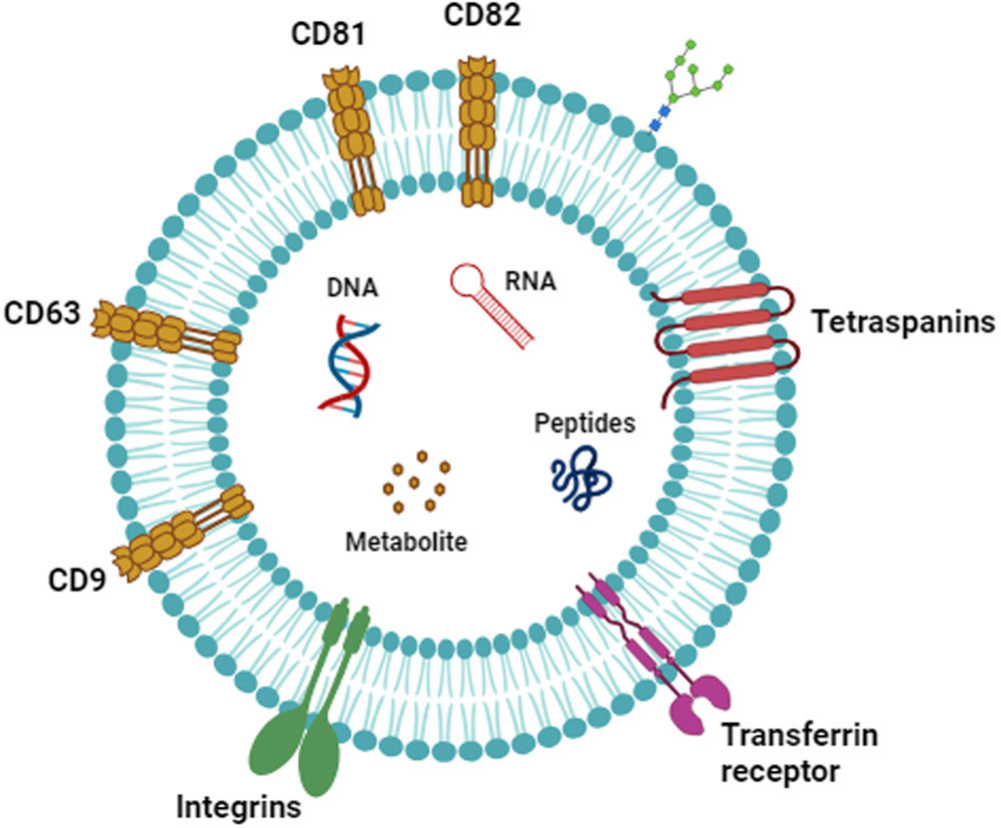
Bioactive molecules of exosomes. Exosomal components include DNA, RNA, peptides, and metabolites. This image was created using BioRender.

The structure of exosomes is characterized by a nanospherical membrane composed of a lipid bilayer that encapsulates a complex biological cargo. Predominantly, exosomes contain proteins, lipids, and various nucleic acids, encompassing DNA, mRNA, miRNA, and other noncoding RNA species (Figure 1) (Jeppesen et al. 2019; Van Niel et al. 2018). One group of proteins commonly found in exosomes is tetraspanins such as CD9, CD63, CD81, and CD82, which serve as markers for exosome identification (Jeppesen et al. 2019; Mathieu et al. 2021). These proteins are involved in membrane organization, exosomal cargo loading, sorting, and production that facilitate intercellular communication (Gauvreau et al. 2009). Additionally, exosomes, being derived from the intracellular endosomal compartment, contain membrane transport and fusion proteins such as GTPases, flotillin, Annexin, and Rab family proteins, which are involved in the assembly, trafficking of exosomes, and fusion events (Van Niel et al. 2018; Phuyal et al. 2014). Heat shock proteins (Hsp70 and Hsp90) are also notable constituents of exosomes, participating in protein folding, stabilization, and cellular stress response. The specific composition of proteins in exosomes can vary depending on the cell type and physiological conditions (Ha et al. 2016; Vlassov et al. 2012). Tetraspanins, a group of transmembrane proteins, within exosomes play regulatory roles in diverse cellular processes, including cell adhesion, fusion, migration, and signaling (Kooijmans et al. 2012). Integrins, another class of adhesion molecules, are also abundantly present in exosomes. These proteins play a crucial role in facilitating cellular attachment to the extracellular matrix and are involved in various cellular processes, including vesicle adhesion to target cells. Notably, Integrins on the surface of exosomes can interact with ligands present on recipient cells, enabling the attachment and subsequent uptake of exosomes by target cells (Clayton et al. 2004; Rieu et al. 2000). Alongside tetraspanins, other proteins associated with exosomes include thrombospondin, lactadherin, CD55, CD59, TSG101, and ALIX (Colombo et al. 2013; Kooijmans et al. 2012; Abrami et al. 2013; Larios et al. 2020). The incorporation of these various proteins into exosomes during their production helps facilitate intercellular signaling and cargo transport, contributing to the functional roles of exosomes in cell‐to‐cell communication and physiological processes (Kalani et al. 2014).

Exosomes not only contain proteins but also include a wide variety of lipids, and the specific lipid composition of exosomes can vary depending on the exosome type. Some of the major lipid components found in exosomes include sphingomyelin, monosialodihexosylganglioside (GM3), phosphatidylinositol, phosphatidylcholine, phosphatidylethanolamine, and phosphatidylserine (Subra et al. 2007; Kajimoto et al. 2013). Sphingomyelin and GM3 are particularly important in determining the stiffness or rigidity of exosomes. These lipids contribute to the physical properties of the exosome membrane and influence its ability to interact with other cells or structures (Vlassov et al. 2012; Piccin et al. 2007). Phosphatidylserine exposure is implicated in various cellular processes, including recognition and clearance of apoptotic cells, intercellular signaling, and immune regulation (Piccin et al. 2007). Additional lipid constituents with the potential to be present in exosomes comprise cholesterol, phosphoglycerides, ceramides, and saturated fatty acid chains (Trajkovic et al. 2008). The diverse lipid composition of exosomes assumes a critical role in shaping their structure, stability, and functionality (Jeppesen et al. 2019).

In the male reproductive system, a proteomic analysis identified 1474 proteins in human seminal exosomes. Gene ontology analysis revealed that these seminal exosome proteins are predominantly associated with exosomes, cytoplasm, and cytosol, participating in processes such as energy pathways, cell growth, and transport (Yang et al. 2017). It is noteworthy that each segment of the epididymis generates a varied population of epididymosomes, each exhibiting distinct lipid and protein compositions (Girouard et al. 2011; Nixon et al. 2019; Frenette et al. 2010). The proteins linked to epididymosomes define two separate vesicle groups: the first, rich in CD9 and other tetraspanin proteins, interacts with viable spermatozoa; the second, identified by the presence of the epididymal sperm binding protein 1 (ELSPBP1), interacts with dying or dead spermatozoa (D'amours et al. 2012). Epididymosomes transport a substantial payload of proteins to spermatozoa, comprising vital classes of enzymes, chaperones, and structural proteins. For instance, cysteine‐rich secretory protein 1 (CRISP1) (Hu et al. 2018), sperm adhesion molecule 1 (SPAM1 or PH‐20) (Martin‐DeLeon 2006), and macrophage migration inhibitory factor (MIF) (Eickhoff et al. 2004). Additionally, epididymosomes carry proteins that offer protection against microbial and proteolytic assaults (Sullivan and Saez 2013), and Glutathione peroxidase (GPX) enzymes that prevent premature acrosome reactions and protect sperm from oxidative stress (Chabory et al. 2009). As epididymosomes traverse from the caput to the cauda epididymis, they undergo alterations in their lipid composition, marked by an increase in sphingomyelin levels and a decrease in cholesterol content (Rejraji et al. 2006).

Prostasomes are the predominant exosomes found in seminal plasma. These exosomes can be categorized into two distinct populations based on their size and molecular composition: smaller vesicles enriched in glioma pathogenesis‐related 2 (GLIPR2) and larger vesicles enriched in Annexin A1 (ANXA1) proteins (Aalberts et al. 2012). These vesicles contain a variety of components, including enzymes, signal transduction proteins, chaperones, transport and structural proteins, GTP‐binding proteins, and other signaling molecules (Poliakov et al. 2009). In addition, they are enriched with high levels of prostatic‐specific acid phosphatase (ACP3 or ACPP, also known as PAP), prostate‐specific antigen (KLK3 or PSA), type 2 transmembrane serine protease (TMPRSS2), prostate‐specific transglutaminase (TGM4), and prostate stem cell antigen (PSCA) (Poliakov et al. 2009; Ronquist 2015). Furthermore, prostasomes have been shown to possess the ability to generate adenosine triphosphate (ATP), implying their involvement in energy metabolism (Ronquist et al. 2013).

Proteins present in prostasomes display antioxidant, antimicrobial, and antibacterial properties. Their coagulant properties function to obstruct sperm contact with female blood, shielding spermatozoa from the immune response within the female reproductive tract by inhibiting the phagocytosis of sperm by monocytes and neutrophils (García‐Rodríguez et al. 2018; Szczykutowicz et al. 2019). The protein cargoes within prostasomes are transferred to spermatozoa just like epididymosomes. They play a critical role in sperm survival and motility through calcium‐dependent signaling pathways (Andrews et al. 2015). The lipid composition of prostasomes is characterized by a predominance of saturated or monounsaturated fatty acids. Notably, the enhanced stability and rigidity of the prostasomal membrane can be attributed to a significantly high concentration of cholesterol and sphingomyelin (Sullivan and Saez 2013).

The presence of nucleic acids, including miRNAs, mRNAs, and small noncoding RNAs (sncRNAs), in exosomes suggests that exosomes can serve as carriers of genetic information between cells, providing a mechanism for intercellular communication and the transfer of regulatory molecules. The transfer of nucleic acids through exosomes has the potential to modulate gene expression and influence cellular functions in recipient cells (Hessvik and Llorente 2018). Altered patterns of miRNA expression have been observed in infertile patients. Examination of the small RNAs (sRNAs) profile has indicated that exosomes derived from ejaculate selectively retain various types of sncRNAs, with miRNAs being the most abundant, followed by YRNAs, ribosomal RNA (rRNA) fragments, tRNA fragments (tRFs), sncRNAs derived from protein‐coding genes, and piRNAs, in decreasing order of abundance (Vojtech et al. 2014). The sRNAs cargo within epididymosomes has been analyzed and identified a wide array of sRNA species, encompassing miRNAs, tRFs, as well as sRNAs originating from small nuclear RNAs (snRNAs), small nucleolar RNAs (snoRNAs), and rRNAs (Hutcheon et al. 2017). Seminal exosomes exhibit a distinct profile of miRNAs that significantly differs from those found in other biological fluids (Vojtech et al. 2014). The RNA content, particularly miRNA, of exosomes in semen appears to vary depending on the cell of origin, which allows it to reflect the pathophysiological conditions of the originating organ. This variability is significant when considering miRNA expression profiles in exosomes as potentially reliable biomarkers with significant implications for diagnostic and therapeutic applications (Barceló et al. 2018). Epididymosomes play a role in modulating the epigenome of spermatozoa during their journey through the epididymis. These vesicles have the ability to transport sRNAs cargo to spermatozoa (Reilly et al. 2016). Limited evidence exists regarding the transfer of the nucleic acids encapsulated in prostasome to the spermatozoa, including DNA, coding RNAs, and regulatory RNAs with potential modulatory functions.

## 3 Cross‐Species Evidence: Relevance and Limitations

Due to the scarcity and incompleteness of human data on seminal exosomes, evidence from animal models was incorporated to provide comparative insights. While such data are valuable, extrapolating findings from animals to human reproductive physiology must be approached with caution, as differences in gene expression patterns, regulatory networks, and epigenetic mechanisms can lead to species‐specific variations in exosome function and infertility‐related processes (Cardoso‐Moreira et al. 2020; Cain et al. 2011). Hence, mechanistic findings in animals should be interpreted as plausible models rather than definitive evidence of identical effects in humans. Despite these limitations, animal studies remain highly informative. Comparative investigations indicate that several fundamental aspects of seminal exosomes biology—including biogenesis pathways, structural markers, and broad functional roles—are remarkably conserved across mammals. These include key tetraspanins (CD9, CD81, CD63, CD82), consistently identified in both human and animal seminal exosomes, and act as critical regulators of vesicle biogenesis, cargo sorting, and signal transduction (Thimon et al. 2008; Nixon et al. 2019; Jankovičová et al. 2020; Zhang, Vos, et al. 2020; Du et al. 2016; Alvarez‐Rodriguez et al. 2019; Barranco et al. 2019). Other exosomal marker proteins, including Alix and TSG101, have also been identified in human (Yang et al. 2016), buffalo (Yu et al. 2023), and mice (Batra et al. 2025). Their widespread presence supports cross‐species comparability and provides a mechanistic framework for exploring the potential roles of seminal exosomes in human reproduction. Moreover, several molecular cargos are shared across species, including proteins such as P25b/P26h (Légaré, Bérubé, et al. 1999; Frenette and Sullivan 2001; Légaré, Gaudreault, et al. 1999; Gaudreault et al. 2001), SPAM1/PH‐20 (Martin‐DeLeon 2006; Griffiths et al. 2008; Morin et al. 2005; Sabeur et al. 1997), CRISP1 (Koppers et al. 2010; Krätzschmar et al. 1996; Cohen et al. 2011; Maldera et al. 2011), MIF (Eickhoff et al. 2004; Frenette et al. 2003, 2005). A proteomic study by Ronquist et al. identified 30 proteins present in prostasomes from human, bull, horse, and dog (Ronquist et al. 2013). Exosomal miRNA families, including let‐7, miR‐21, miR‐10, miR‐26, miR‐30, and miR‐34, have also been identified in humans (Barceló et al. 2018; Vojtech et al. 2014; Zhang et al. 2025), mice (Sharma et al. 2018), pigs (Chen et al. 2017), and boars (Dlamini et al. 2023; J. Sun et al. 2021), although their regulatory roles may vary between species. We emphasize that such cross‐species presence should be interpreted as evidence of possible shared pathways rather than direct proof of functional equivalence.

Finally, combined evidence from animal and human studies supports several conserved biological functions of seminal exosomes, including regulation of sperm maturation and motility in human (Murdica, Giacomini, et al. 2019), boars (Du et al. 2016; Guo et al. 2019), bovine (Frenette et al. 2003), mouse (Chen et al. 2006), and pig (Zhao et al. 2024); induction of capacitation and acrosome reaction in boar (Piehl et al. 2013), human (Pons‐Rejraji et al. 2011; Palmerini et al. 2003), and rat (Dong et al. 2021); facilitation of fertilization and embryo development in mouse (Chen et al. 2006; Park et al. 2011), human (Lal et al. 2022), bovine (Caballero et al. 2012), and pig (Chen et al. 2020); and modulation of immune responses in the female reproductive tract in human (Robertson and Sharkey 2016; Rooney et al. 1993; Paktinat et al. 2019), pig (Bai et al. 2018), and mice (D. Wang et al. 2021). These parallels, while not definitive, provide a mechanistic framework for interpreting the potential roles of seminal exosomes in human reproduction.

## 4 Seminal Exosomes and Male Fertility

**Figure 2:**
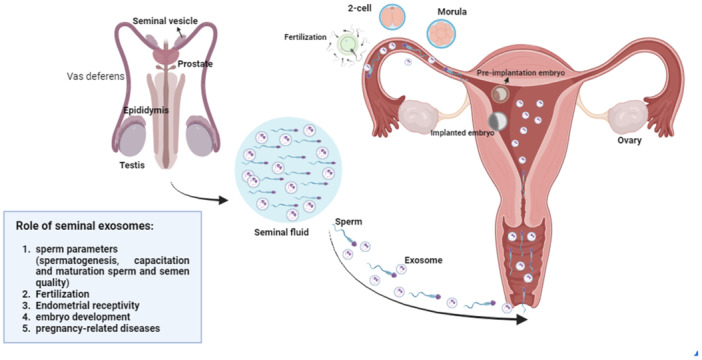
Production and function of seminal exosomes in reproductive systems. Exosomes originate from various organs in the male reproductive system. Seminal exosomes play a significant role in sperm production, maturation, sperm parameters, fertilization, preimplantation, embryo implantation, and endometrial receptivity. This image was created using BioRender.

Seminal exosomes play a crucial role in facilitating communication between seminal plasma and spermatozoa, positively impacting essential processes related to sperm functions (Baskaran et al. 2020). They are involved in the transport and delivery of various bioactive molecules and signaling factors to the developing sperm cells. The proteins present in seminal exosomes have diverse functions and are involved in the growth and maintenance of cells, energy pathways, and protein metabolism (Yang et al. 2017). They contribute to cellular processes such as signal transduction, cell adhesion, membrane trafficking, and enzymatic activities (Yang et al. 2017; Martin‐DeLeon 2015). A growing body of research indicates that exosomes originating from the male genital tract, including Sertoli cells, epididymis, and prostate, play key roles in germ cell development, sperm maturation, and fertilization outcomes (Figure 2) (Sullivan 2015; Tian et al. 2024; Conine et al. 2018).

### 4.1 Seminal Exosomes and Spermatogenesis

Spermatogenesis in mammals is primarily governed by Sertoli cells, the key somatic cell population, providing physical support and maintaining a stable microenvironment for male germ cells (Wong and Khan 2020). Beyond their structural role, animal studies have demonstrated that Sertoli cell‐derived exosomes regulate germ cell proliferation, differentiation, and apoptosis (Q. Li et al. 2021; Dashti et al. 2025). These vesicles, secreted via paracrine mechanisms, are readily taken up by spermatogonial stem cells (SSCs), enabling dynamic communication between Sertoli cells and germ cells—an interaction essential for sustaining spermatogenic efficiency (Ruthig and Lamb 2022; Meroni et al. 2019). Sertoli cell‐derived exosomes are enriched with bioactive proteins and nucleic acids that regulate SSCs function (Tian et al. 2024; Q. Li et al. 2021; Gao et al. 2023). Notably, several miRNAs have emerged as key mediators of this regulation. For instance, miR‐210‐3p inhibits spermatogonial apoptosis, thereby enhancing germ cell survival and spermatogenesis. Its presence in seminal exosomes in a rat model has been proposed as a diagnostic biomarker for Sertoli cell injury in conditions such as varicocele (Ma et al. 2021). Similarly, miR‐486‐5p, significantly enriched in mice Sertoli cell exosomes, is transferred to SSCs where it downregulates PTEN, upregulates STRA8 and SYCP3, and promotes SSC differentiation. Inhibition of its transfer disrupts SSCs differentiation, underscoring its critical signaling role (Q. Li et al. 2021). Moreover, in mice, Sertoli cell‐derived exosomal miR‐30a‐5p targets Zeb2 in SSCs, regulating Fgf9 ubiquitination and activating the mitogen‐activated protein kinase (MAPK) pathway to promote SSC proliferation and differentiation (Gao et al. 2023). Other miRNAs, including miR‐493‐5p, regulate glial cell line‐derived neurotrophic factor (GDNF) production by targeting Gdnf mRNA in mice, thereby modulating the SSC niche (Tian et al. 2024).

Beyond germ cells, Sertoli cell exosomes also influence Leydig cell physiology. In vitro studies demonstrate that rat Sertoli cell exosomes enhance Leydig cell survival compared with untreated controls (Ma et al. 2022). In vivo injection of these exosomes into rat testes confirmed their uptake by Leydig cells and revealed increased expression of Ccl20 and activation of the AKT signaling pathway, indicating a prosurvival effect (Ma et al. 2022). Moreover, specific exosomal miRNAs play regulatory roles in Leydig cell steroidogenesis. In a mouse model, miR‐145‐5p has been identified as a suppressor of steroidogenic factor 1, leading to reduced expression of steroidogenic genes, lipid droplet accumulation, and impaired testosterone production, thereby suggesting its potential as a biomarker for developmental hypogonadism (Liang et al. 2021). Sertoli cell‐derived exosomes transfer miR‐9‐3p to Leydig cells, where it targets the STAR gene and suppresses testosterone biosynthesis, as demonstrated in a mouse study (Huang et al. 2022). Collectively, these findings highlight the multifaceted role of Sertoli cell‐derived exosomes in coordinating both germ cell development and Leydig cell function. Because exosome‐specific data from human Sertoli cells are limited, we included animal studies. Human evidence mostly derives from mixed EVs in Sertoli cell line studies (Tan et al. 2022; Ma et al. 2023), whereas direct Sertoli exosome data are available in animal models.

### 4.2 Seminal Exosomes and Sperm Maturation

Sperm produced in the seminiferous tubules of the testes are initially immotile and incapable of fertilizing an oocyte upon entering the epididymis. Posttesticular maturation in the epididymis is therefore essential, providing a conducive environment for spermatozoa to acquire motility and fertilization competence (Stival et al. 2016). This maturation involves dynamic interactions between sperm and the epididymal intraluminal compartment, which facilitate macromolecular modifications critical for sperm function (Cornwall 2009; Zhou et al. 2018).

During epididymal transit, sperm undergo profound physiological and biochemical transformations, including changes in protein content, cholesterol, phospholipids, and sncRNA cargo (Nixon et al. 2019; Sullivan 2015; Joshi et al. 2023; Hong et al. 2021; Sullivan et al. 2018). These changes are largely mediated by epididymal exosomes (epididymosomes), which deliver proteins, RNAs, and lipids to compensate for the transcriptional inactivity of spermatozoa (Sullivan and Saez 2013; Martin‐DeLeon 2015), thereby regulating sperm–cell interactions and supporting their proper development and storage across multiple species (Girouard et al. 2011; Nixon et al. 2019; Rejraji et al. 2006; Conine et al. 2018), including humans (Barceló et al. 2018; Thimon et al. 2008; Yang et al. 2017). Based on the epididymal region from which exosomes originate, the cargo carried by them is integrated into sperm and transported to specific regions of the sperm regions (Rejraji et al. 2002; Frenette et al. 2010; Barrachina et al. 2022). Much of the direct evidence from animal models, including mice and bulls, shows that epididymosomes display segment‐specific lipid profiles, with differences in cholesterol, phospholipids, sphingomyelin, and polyunsaturated fatty acids between those from the proximal region and the cauda epididymis, reflecting dynamic lipid modifications during epididymal transit (Girouard et al. 2011; Rejraji et al. 2006). Moreover, proteomic analyses in mice revealed that 146 proteins differed between caput and corpus epididymosomes, while 344 proteins varied between corpus and cauda segments (Nixon et al. 2019). Similar analyses in bulls identified 555 proteins in caput‐derived and 438 in cauda‐derived epididymosomes, with only 231 shared proteins (Girouard et al. 2011). Gene ontology analysis revealed the functions of the identified proteins, categorizing them as enzymes, transporters, structural proteins, and chaperones, and emphasizing the critical role of these vesicles in sperm maturation and function (Girouard et al. 2011; Nixon et al. 2019). Proteomic analyses of epididymosomes have identified several significant proteins that are directly implicated in sperm functionality, including CRISP1 regulates Ca²⁺ channels in the sperm membrane, influencing sperm function (Hu et al. 2018; Krätzschmar et al. 1996; Maldera et al. 2014; Weigel Muñoz et al. 2019), while SPAM1/PH‐20 is involved in sperm–zona pellucida adhesion (Martin‐DeLeon 2006; Sullivan and Saez 2013; Griffiths et al. 2008; Evans et al. 2003). The cytokine MIF, often enriched alongside aldose reductase (AKRB1) in CD9‐positive epididymosomes, plays a role in sperm motility acquisition by contributing to sperm motility through the polyol pathway of glucose metabolism (Eickhoff et al. 2004; Frenette et al. 2003, 2006). In bulls, tetraspanins such as CD9 and CD26, together with MIF, are incorporated into the sperm flagellar fibrous sheath during epididymal transit, further supporting motility and maturation (Girouard et al. 2011; Sullivan 2015; Caballero et al. 2013). Beyond protein delivery, epididymosomes influence the sperm epigenome by transferring RNAs. In animal models, deep sequencing analyses after in vitro co‐incubation of sperm with epididymosomes show widespread incorporation of sRNAs, underscoring their role in shaping the regulatory RNA profile of sperm during posttesticular maturation (Hutcheon et al. 2017; Reilly et al. 2016; Sharma et al. 2018, 2016; Belleannée et al. 2013).

During ejaculation, sperm encounter prostate‐derived exosomes, or prostasomes, which are enriched in divalent cations (Ca²⁺, Zn²⁺, Mg²⁺) and modulate sperm ion concentrations and flagellar motility via a pH‐dependent fusion mechanism (Palmerini et al. 2003). Prostasome recruitment is highly specific, occurring primarily at the sperm head under neutral pH and bicarbonate conditions typical of the female reproductive tract (Aalberts et al. 2013, 2014). Near the oocyte‐cumulus complex, prostasomes could fuse with sperm cells, delivering cytosolic and membrane components that influence capacitation, motility, and the acrosome reaction (del Carmen Neri‐Vidaurri et al. 2006; Vyas et al. 2019; Vickram et al. 2020).

Collectively, these findings illustrate a continuum of EV‐mediated regulation, beginning with epididymosomes that remodel sperm during epididymal transit, and extending to prostasomes that fine‐tune sperm function during ejaculation and capacitation. This integration highlights the central role of exosomes as dynamic carriers of proteins, RNAs, and signaling molecules, orchestrating molecular and functional maturation of sperm in a highly coordinated, stepwise manner.

### 4.3 Seminal Exosomes and Sperm Capacitation

During posttesticular maturation, sperm undergo significant lipid modifications and notable changes in their proteome environment (Watanabe et al. 2017; Hall et al. 1991; S. Chen et al. 2021). These changes are crucial in preparing sperm for capacitation as they traverse the female reproductive tract, aiming to enhance the stability of the sperm plasma membrane, which is essential for long‐term sperm preservation. Capacitation involves a coordinated series of structural and functional alterations, including modifications in sperm membrane composition, enzymatic activity regulation, and protein phosphorylation. A hallmark of capacitation is the hyperactivation of spermatozoa, characterized by asymmetrical flagellar beating and a rise in cytoplasmic Ca²⁺ (Ferramosca and Zara 2014; Chang and Suarez 2011; López‐González et al. 2014). To ensure successful fertilization, capacitation must be precisely timed to occur in synchrony with ovulation (Tao et al. 2017). After ejaculation, components of seminal plasma—particularly decapacitation factors—coat and stabilize the sperm surface to prevent premature capacitation (Pons‐Rejraji et al. 2011; Kawano et al. 2008). Prostasomes complement this process by transferring cholesterol and sphingomyelin to the sperm membrane, thereby maintaining stability and delaying the acrosomal reaction (García‐Rodríguez et al. 2018). This inhibition is largely achieved by maintaining the cholesterol‐to‐phospholipid ratio within the sperm membrane, since capacitation involves the removal of cholesterol from the membrane (Begley and Quinn 1982).

As spermatozoa advance through the female reproductive tract, capacitation initiates a cascade of signaling events that includes glycoprotein removal, phospholipid redistribution, and activation of bicarbonate‐sensitive adenylate cyclase (sACY). This activation increases intracellular cyclic adenosine monophosphate (cAMP) and drives phosphorylation at serine, threonine, and tyrosine residues (Bailey 2010; Puga Molina et al. 2018; Candenas et al. 2018). Seminal exosomes are now recognized as central players in this regulatory process, providing molecular cargo that fine‐tunes capacitation‐related events, as demonstrated by several studies (Murdica, Giacomini, et al. 2019; Bechoua et al. 2011). Seminal plasma exosomes mediate the prevention of premature capacitation through various mechanisms, such as inhibition of cholesterol efflux, reduction in fluidity of sperm apical membranes, regulation of acrosome reaction, and acquisition of Ca^2+^ signaling tools (Park et al. 2011; Aalberts et al. 2014; Berruti and Paiardi 2011; Zhang, Song, et al. 2020). They modulate tyrosine phosphorylation patterns, a critical intracellular signaling event controlling capacitation (Bechoua et al. 2011), and transfer proteins that interact directly with sperm to inhibit cholesterol efflux (Piehl et al. 2013; Pons‐Rejraji et al. 2011). In boars, seminal plasma exosomes have been shown to deliver decapacitation factors such as spermadhesin (AWN) and porcine seminal plasma protein‐1 (PSP‐1) to sperm, thereby reinforcing their inhibitory role in preventing premature capacitation. These vesicles in seminal plasma can interact with spermatozoa and prevent premature activation, preserving sperm viability and functionality until they reach the site of fertilization (Du et al. 2016). In rats, palmitoylated GLB1L4 is expressed in the epididymal caput, packaged into epididymosomes, and transferred to spermatozoa—knockdown of GLB1L4 impairs capacitation and reduces fertility (Dong et al. 2021; Zhen et al. 2009)—however, direct evidence for this mechanism in humans is not yet available. Prostasomes, another population of seminal exosomes, amplify this process by elevating cAMP levels (Pons‐Rejraji et al. 2011), and supporting ion channel activation, which leads to membrane hyperpolarization, pH elevation, and Ca²⁺ channel opening (Candenas et al. 2018). Under physiological conditions, elevated progesterone levels promote the fusion of prostasomes with the sperm membrane (Palmerini et al. 2003; Aalberts et al. 2014), facilitating the transfer of prostasome‐derived enzymes, receptors, and annexins that sustain Ca²⁺ signaling. Increased sperm responsiveness to cumulus cell–secreted progesterone ultimately triggers the acrosome reaction (Park et al. 2011; Aalberts et al. 2014; Prajapati et al. 2022; Jin et al. 2011).

## 5 Seminal Exosome and Sperm Parameters

Recent findings, along with evidence showing the uptake of exosomes by spermatozoa (Murdica, Giacomini, et al. 2019; Park et al. 2011), emphasize the significant impact of seminal exosomes on influencing sperm characteristics and functionality. Consequently, the compounds enclosed within seminal plasma exosomes and the mechanisms by which exosomes shuttle these compounds for intercellular communication have become focal points of research interest. Although seminal plasma exosomes obtained from various individuals exhibited similarities in terms of size, shape, and exosomal markers, their contents differed significantly (Murdica, Giacomini, et al. 2019). This highlights the diversity of cargo carried by seminal exosomes, underscoring their potential role in mediating diverse physiological effects on sperm cells and within the reproductive microenvironment.

Testosterone deficiency can induce alterations in the miRNAs present in seminal exosomes, which, in turn, transfer these alterations to spermatozoa. The changes in the miRNA content of seminal exosomes and sperm from adult pigs that underwent prepubertal hemicastration were detected. A total of 16 miRNAs were identified as differentially expressed between the spermatozoa from prepubertal hemicastrated pigs and the healthy pigs. The miRNAs that were found to be differentially expressed in spermatozoa from prepubertal hemicastrated pigs were enriched in apoptosis‐associated signaling pathways including MAPK pathway, p53 pathway, and mammalian target of rapamycin (mTOR) pathway. It appears that these alterations in the sperm miRNAome are responsible for the decrease in sperm motility observed in hemicastrated pigs (Ma et al. 2018). It is believed that specific miRNAs regulate genes involved in apoptosis and key signaling pathways associated with fertility and sperm function (Yang et al. 2017; Belleannée et al. 2013; Ma et al. 2018).

Semen quality can be affected by male tract exosomes (Murdica, Cermisoni, et al. 2019; Sullivan and Saez 2013; García‐Rodríguez et al. 2018; Piehl et al. 2013). In this regard, the impact of exosomes on spermatozoa was evaluated by assessing progressive motility and capacitation, with the latter being determined through tyrosine phosphorylation and the acrosome reaction (Thimon et al. 2008; Aalberts et al. 2013). Isolation and characterization of seminal exosomes from men with normozoospermic samples, severe asthenozoospermia, and postvasectomy azoospermic revealed that the exosomes obtained from these different groups exhibited similar characteristics in terms of shape, size, expression of markers, and protein expression involved in sperm maturation and fertility potential. Exosomes derived from normozoospermic men have been found to enhance sperm motility and promote capacitation. This effect appears to be attributed to the transfer of CRISP‐1 from exosomes to spermatozoa after ejaculation (Murdica, Giacomini, et al. 2019).

The proteomic profiles of exosomes isolated from the seminal plasma of normozoospermic and severe asthenozoospermic men revealed differences in their protein content that could potentially impact sperm characteristics (Murdica, Cermisoni, et al. 2019; García‐Rodríguez et al. 2018). It was observed that there is a total of 2138 proteins in both groups. Furthermore, the comparative analysis of seminal exosomes proteins showed that 89 proteins were differentially expressed in exosomes derived from normozoospermic samples versus asthenozoospermic samples, of which 37 increased in normozoospermic samples and 52 increased in asthenozoospermic samples. These differential protein expression patterns may contribute to the distinct characteristics of exosomes derived from normozoospermic and asthenozoospermic samples, potentially influencing sperm function and quality in these individuals (Murdica, Cermisoni, et al. 2019). Approximately one‐third of the proteins overexpressed in exosomes derived from normozoospermic samples were involved in reproductive processes. These proteins likely play important roles in supporting sperm function and promoting fertility. Conversely, the highly expressed proteins in exosomes from asthenozoospermic samples did not exhibit specific functional relevance to reproductive processes. This suggests that the altered protein composition in exosomes of asthenozoospermic individuals may not contribute directly to improving sperm motility and capacitation, indicating a potential functional difference in the exosomes derived from normozoospermic and asthenozoospermic individuals (Murdica, Cermisoni, et al. 2019).

In males with unilateral varicocele and infertility, perturbations in exosome‐related protein expression have been detected. The analysis of seminal plasma protein profiles revealed that a total of 47 proteins were differentially expressed in infertile patients with unilateral varicocele compared with fertile men. Among the altered proteins, several exosomal proteins including Annexin A2 (ANXA2), Transferrin (TF), Kinesin‐1 heavy chain (KIF5B), and semenogelin 1 (SEMG1) were found to be consistently expressed differently in the seminal plasma of patients with unilateral varicocele. Based on these findings, it has been proposed that KIF5B and ANXA2 could be considered potential biomarkers for exosomal dysfunction in patients with varicocele. Additionally, the accumulation of angiotensinogen (AGT) in the seminal plasma may be predictive of sperm motility impairment and higher levels of DNA fragmentation in spermatozoa from infertile individuals with unilateral varicocele (Panner Selvam et al. 2021). These findings underscore the link between aberrant seminal plasma proteins expression and disruptions in physiological equilibrium that could impact the fertilization capacity of spermatozoa, emphasizing the significance of comprehending the role of exosome‐associated proteins in male infertility associated with varicocele.

Research has shown that Sertoli cell‐derived exosomes have the ability to traverse the blood‐testis barrier (BTB) and enhance Leydig cell viability in rat model (Ma et al. 2022). In experimental rat models of grade II and III varicocele, an upregulation of miR‐210‐3p has been observed in seminal exosomes. This upregulation is inversely correlated with sperm count and the expression of seminal Inhibin‐B. Exosomal miR‐210‐3p induced by hypoxia serves as an indicator of Sertoli cell functionality in the context of varicocele. Following microsurgical varicocelectomy, a significant reduction in miR‐210‐3p levels has been reported (Ma et al. 2021).

Seminal plasma exosomes have emerged as an alternative avenue for intercellular communication, facilitating the transfer of their molecular cargo to sperm cells (Simons and Raposo 2009). The extracellular adenosine triphosphate (exATP) cargo contained within seminal exosomes plays a pivotal role in regulating boar sperm motility and mitochondrial metabolism. Experimental exposure of boar sperm to exosomal exATP during incubation resulted in enhanced progressive motility, reduced sperm apoptosis, and improved overall viability, indicating a protective effect on sperm function (Guo et al. 2019). Furthermore, exATP increased the concentration of intracellular ATP (inATP) within the sperm, indicating improved energy production, and reduced the ADP/ATP ratio in spermatozoa, suggesting higher energy levels within the sperm. Exposure to exATP resulted in a significant increase in mitochondrial membrane potential in treated spermatozoa, consistent with Ser21 phosphorylation of glycogen synthase kinase‐3 alpha (GSK3α), which acts as a negative regulator of sperm motility and can be inactivated through Ser21 phosphorylation. Interestingly, the content of lactate in the incubation medium decreased upon exATP treatment. Lactate serves as a support for sperm motility and mitochondrial function. However, the dehydrogenase activity of lactate within the sperm increased. Then, exATP promotes the production of lactate, which, in turn, contributes to the generation of inATP to sustain sperm motility (Guo et al. 2019). Human studies have provided compelling evidence that GSK3 inhibits sperm motility (Park et al. 2023; Freitas et al. 2019), and in mice has shown that proteins involved in the Wnt signaling pathway, delivered via exosomes, mediate sperm maturation and the acquisition of motility (Koch et al. 2015; De Robertis and Ploper 2015).

In buffalo, the influence of seminal exosomes on the regulation of sperm maturation and motility was explored in individuals with varying levels of sperm motility (high or low motility) (Yu et al. 2023). The proteomic analysis of the protein association between spermatozoa, seminal plasma, and seminal plasma exosomes in buffalo revealed that 41.8% of exosomal proteins were also detected in spermatozoa. Similarly, 40.7% of the exosomal proteins were detected in the seminal plasma. Approximately 27.6% of the exosomal proteins were found to be present in both seminal plasma and spermatozoa. These proteins may play an essential role in the microenvironmental balance during spermatozoa maturation. According to comparative proteomic analyses, 119 downregulated and 41 upregulated proteins were identified in seminal plasma exosomes from semen samples with low sperm motility compared with those with high sperm motility (Yu et al. 2023). These differentially expressed proteins (DEPs) may be involved in the regulation of sperm motility and could potentially serve as biomarkers for assessing sperm quality and fertility in buffalo. According to Gene Ontology analysis, the majority of the DEPs were associated with hydrolase and metalloproteinase activities. These proteins are likely involved in processes such as sperm‐oocyte recognition, binding of sperm to the zona pellucida, fertilization, and single fertilization events. Moreover, based on Kyoto Encyclopedia of Genes and Genomes (KEGG) analysis, the DEPs were found to be involved in signaling pathways including PPRP, calcium, and cAMP signaling pathways (Yu et al. 2023). These pathways are essential for mediating cellular responses to extracellular signals, calcium regulation, and sperm function.

Seminal plasma exosomes could protect spermatozoa from chilling injuries. Adding human seminal exosomes to the sperm diluent led to an improvement in sperm motility and total antioxidant capacity (TAC) compared with using the original semen alone (Du et al. 2016; Mahdavinezhad et al. 2022). Spermatozoa treatment with EVs including exosomes and microvesicles isolated from normozoospermic semen samples before cryopreservation showed the cryoprotectant effects. According to the obtained results through fluorescence microscopy, exosomes and microvesicles were primarily absorbed in the sperm head region. The post‐thawing results showed that the presence of microvesicles or exosomes in the diluent can improve sperm morphology, motility, and viability compared with untreated samples. There was a significant decrease in the reactive oxygen species (ROS) levels, leading to a subsequent reduction in DNA damage in the post‐thaw spermatozoa. Although the addition of microvesicles or exosomes improved the TAC activity and mitochondrial membrane potential (MMP), the levels of malondialdehyde (MDA), a marker of lipid peroxidation and apoptosis in the post‐thaw spermatozoa, remained unchanged (Mahdavinezhad et al. 2022). The impact of exosomes derived from boar seminal plasma on sperm function during long‐term storage at low temperatures specifically revealed their protective effects against chilling injuries. Exosomes in diluent improved survival times for the sperm and maintained the integrity of the spermatozoa's plasma membrane. This preservation of membrane integrity was attributed to the direct binding of exosomes to the sperm head membrane, which enhanced the stability of the membrane structure. Additionally, the study demonstrated that exosomes played a role in enhancing the sperm's antioxidation properties by maintaining the integrity of the sperm membrane (Du et al. 2016).

## 6 Regulation of Fertilization and Preimplantation Processes by Seminal Exosomes

Fertilization is a complex process involving a series of synchronized molecular events that lead to the fusion of a sperm and an oocyte (Georgadaki et al. 2016). Indeed, the function of the male gamete extends beyond the transportation of genetic material, as it also acts as a signaling agent that interacts with female reproductive tissues to promote fertilization (Saint‐Dizier et al. 2020; Schjenken and Robertson 2020). In the female genital tract, sperm cells undergo a diverse range of biochemical and biophysical changes to acquire fertilization properties and reach the oocyte, underscoring the intricate molecular dynamics essential for successful conception (Mayorga et al. 2007). The ability of seminal exosomes to deliver molecules to both sperm cells and immune cells within the female reproductive tract is what makes them versatile in sperm physiology. This adaptability allows exosomes to influence the molecular composition and behavior of sperm cells, ultimately enhancing their fertilizing capacity (Yáñez‐Mó et al. 2015). Most of the proteins involved in this process bind to the surface of spermatozoa via epididymosomes and prostasomes. They play important roles in sperm maturation and modulate sperm interactions with the female reproductive tract that contribute to the functional development and readiness of sperm cells for successful fertilization (Paktinat et al. 2019; Bai et al. 2018; Samanta et al. 2018).

The interaction between prostasomes and sperm cells within the female reproductive tract serves multiple purposes. First, it assists in the navigation of sperm cells toward the oocyte, facilitating their journey to the site of fertilization. Additionally, prostasomes protect sperm cells from the female immune system, ensuring their survival and function during transit toward the ovum. Proteins transferred by prostasomes, such as galectin 3 and CD48, offer protection to sperm cells deposited in the female reproductive tract (Tarazona et al. 2011; Jones et al. 2010; Ronquist 2012). These proteins are involved in modulating immune response pathways, such as complement pathway (Kitamura et al. 1995), lymphocyte proliferation (Kelly 1995), and phagocytosis (Skibinski et al. 1992) within the female reproductive environment, contributing to the safeguarding and functionality of sperm cells during their passage toward fertilization. The contents of seminal plasma exosomes can be considered an indicator of the fertility potential of spermatozoa. It has been demonstrated that exosomes derived from human and animal seminal plasma contribute to the sperm‐egg fusion process through the involvement of specific proteins such as ANXA2, GLIPR2 (Aalberts et al. 2012), Glutathione peroxidase‐5 (GPX5) (Rejraji et al. 2002; Chabory et al. 2009), Kallikrein 2 (KLK2) (García‐Rodríguez et al. 2018), SPAM1 (Martin‐DeLeon 2006; Griffiths et al. 2008; Sabeur et al. 1997), PSA (Aalberts et al. 2014), and Kinesin family member 5B (KIF5B) (Baskaran et al. 2020). These proteins are implicated in various aspects of sperm function and interactions critical for successful fertilization, underscoring the significance of seminal plasma exosomes in influencing the reproductive potential of sperm cells.

SPAM1, also known as PH‐20, is a glycosylphosphatidylinositol (GPI) anchored protein that is transmitted to the sperm plasma membrane via epididymosomes. It plays a crucial role in sperm‐zona pellucida adhesion and serves as a hyaluronidase, facilitating the penetration of sperm through the cumulus layer surrounding the oocyte (Martin‐DeLeon 2006; Griffiths et al. 2008; Sabeur et al. 1997; Chen et al. 2006; Zhang and Martin‐DeLeon 2003). On the other hand, GPX5 is a seleno‐independent glutathione peroxidase with limited enzymatic activity toward hydrogen peroxide. During the transit of spermatozoa through the epididymis, GPX5 is transferred to the acrosomal region of sperm cells. It is thought to help protect sperm from triggering acrosome reactions prematurely, contributing to the maintenance of sperm integrity and functionality during their journey toward fertilization (Baskaran et al. 2020).

P25b/P26h, known as dicarbonyl/L‐xylulosereductase (DCXR) or P34H in humans, is a sperm‐binding protein that is secreted in association with epididymosomes and anchored by GPI. As sperm transit through the epididymis, this protein is incorporated onto their surface. Its primary role is to mediate the binding of sperm to the zona pellucida, a critical step in the fertilization process (Frenette and Sullivan 2001; Légaré, Gaudreault, et al. 1999; Sullivan et al. 2007). Glioma Pathogenesis‐related protein 1 (GliPriL1), a member of the cysteine‐rich secretory protein (CAP) family, is another protein that is GPI‐anchored to the sperm plasma membrane and plays a significant role in fertilization in animals (Caballero et al. 2012; Gaikwad et al. 2019) and in human with a similar potential role (Consortium 2025). Both p25b and GliPriL1 are involved in sperm‐egg interactions, highlighting their importance in the intricate process of fertilization (Légaré, Gaudreault, et al. 1999; Caballero et al. 2013).

Recent studies have revealed that sperm tRNA‐derived small RNAs (tsRNAs) transmit paternal epigenetic information and may function as adaptive epigenetic regulators, influencing offspring phenotypes in response to environmental stress. Additionally, they are capable of regulating early embryonic cleavage stages during preimplantation (Y. Zhang et al. 2018; Chen et al. 2016; X. Chen et al. 2021). Research by Xiaoxu Chen et al. has confirmed the transmission of tsRNAs from semen‐derived epididymosomes to sperm cells. A specific functional group of tsRNAs, referred to as Gln‐TTGs, has been identified that plays a role in the early cleavage of porcine preimplantation embryos. This group likely exerts its influence by regulating genes associated with the cell cycle and retrotransposons (Chen et al. 2020), shedding light on the intricate mechanisms through which tsRNAs probably impact early embryonic development. Not only tsRNAs have been detected in porcine and human seminal plasma (Vojtech et al. 2014; Chen et al. 2017), but they have also been found to be present in mice, where they are notably scarce in the testis but significantly enriched in the epididymis (Sharma et al. 2018, 2016). Studies have shown that in porcine and human epididymal sperm cells, only a minor fraction (approximately 20%) of sRNAs are tsRNAs (Schuster et al. 2016). These observations underscore the diverse distribution and prevalence of tsRNAs across species and within different reproductive contexts, highlighting their potential significance in the regulation of reproductive processes and inheritance mechanisms.

Further analysis of the gene expression variances within exosomes may eventually unveil the impact of distinct signaling pathways linked to positive or negative pregnancy outcomes. Semen samples utilized in Intrauterine Insemination (IUI) have provided compelling evidence that the RNA content of exosomes differs significantly between samples resulting in successful pregnancies and those that did not. An overall increase in expression linked to chromosomes 1, 10, 12, 16, and 21 was detected. Through RNA sequencing, numerous genes were found to be either upregulated or downregulated in sperm cells used in successful IUI procedures compared with unsuccessful attempts. It was detected that miRNAs and lincRNAs levels were decreased in successful IUI cases, while coding mRNAs levels were increased in successful attempts. Moreover, a higher number of RNA copies were associated with the successful IUI group (Lal et al. 2022). These findings shed light on the potential role of exosomal RNA content in predicting and influencing the outcomes of reproductive interventions like IUI.

The immune milieu within the uterus is vital for facilitating successful embryo implantation. Seminal plasma, especially seminal plasma exosomes, undergoes changes with age and can affect the immune microenvironment of uterine in the female mice (Mor et al. 2011). The introduction of seminal plasma exosomes (excluding epididymosomes) from young mice to the aged‐seminal plasma group led to enhancements in the declining embryo implantation rate. This indicates that introducing seminal plasma exosomes from young mice had a positive effect on embryo implantation in the aged‐seminal plasma group. Also, in vitro and in vivo investigations demonstrated that seminal plasma exosomes from aged mice displayed an inhibitory effect on dendritic cell maturation, providing insights into their potential impact on the immune environment. These findings suggest that age‐related changes in seminal plasma exosomes can be partly responsible for the decreased implantation rates observed in the aged‐seminal plasma group compared with the young‐seminal plasma group, potentially mediated through the immunomodulation of the uterus (D. Wang et al. 2021).

## 7 The Impact of Seminal Exosomes on Endometrium During Implantation

Seminal fluid, in addition to serving as a medium for sperm transport to facilitate oocyte fertilization, significantly influences female reproductive tissues. It induces molecular and cellular changes that optimize conditions for conception and support the establishment of a successful pregnancy. The communication between male and female through seminal fluid signaling is observable in various mammalian species, including humans, and the effects of seminal fluid in invertebrates suggest the existence of evolutionarily conserved mechanisms (Schjenken and Robertson 2020). While the impacts of seminal fluid on women are not as extensively studied, clinical observations indicate that exposure to the seminal fluid of the partner can potentially increase the likelihood of a healthy pregnancy (Saint‐Dizier et al. 2020; Schjenken and Robertson 2020). Bioactive components in seminal fluid function as critical signaling molecules, facilitating communication between seminal fluid and the female reproductive system (Paktinat et al. 2019; Bai et al. 2018; Rodriguez‐Caro et al. 2019). Seminal plasma components can directly interact with immune cells within the reproductive tract, stimulating immune‐regulatory and pro‐tolerance functions. One significant outcome of the immune reaction to seminal fluid is the generation of immune suppressive Treg (regulatory T) cells that play a crucial role in establishing adaptive tolerance essential for supporting pregnancy (Robertson et al. 2018). The strength and characteristics of the T cell response are influenced by the balance of signals derived from seminal fluid, leading to modifications in the expression of cytokines and chemokines. These alterations influence the quantity, characteristics, and antigen‐presenting ability of the recruited macrophages and dendritic cells, illustrating the intricate interplay between seminal fluid signals and the immune system in shaping the immune environment in the female reproductive tract (Schjenken and Robertson 2020; Sharkey et al. 2012).

Seminal plasma has the ability to influence human dendritic cells, directing them toward specific functional phenotypes, characterized by a heightened ability to generate interleukin‐10 (IL‐10) and transforming growth factor‐beta (TGF‐β) (Remes Lenicov et al. 2012). This phenotype also facilitates the formation of immunosuppressive regulatory T cells (Treg cells) (Meuleman et al. 2015). When human endometrial epithelial and stromal cells are cultured with seminal plasma in vitro, they undergo transcriptional activation, leading to the secretion of various pro‐inflammatory and chemotactic cytokines (Chen et al. 2014). This process is associated with an increased secretion of IL‐1B, IL‐6, and leukemia inhibitory factor (LIF) (Gutsche et al. 2003). Disrupting seminal plasma signaling in mice through seminal vesicle excision results in a dampening of uterine cytokine production, compromising the immune adaptation necessary for successful pregnancy (Guerin et al. 2011; Johansson et al. 2004; Bromfield et al. 2014).

While decidualization in women occurs autonomously regardless of seminal fluid exposure, in vitro studies indicate that components within seminal plasma can enhance the decidual response. These effects are thought to act directly on endometrial stromal cells and potentially indirectly through interactions with immune cells (Doyle et al. 2012). EVs found in seminal fluid, mainly prostasomes and epididymosomes, are presumed to possess signaling capabilities due to their established roles in fertility and immune modulation (Tannetta et al. 2014; Ronquist 2015; Barrachina et al. 2022; Tarazona et al. 2011), highlighting their significance in the intricate interplay between seminal fluid components and reproductive physiology. Seminal exosomes from both humans and pigs have been shown to play a role in the immuno‐inflammatory responses within the endometrium. They are involved in regulating the uterine microenvironment relevant to embryo implantation by modulating the levels of chemokines and cytokines (Paktinat et al. 2019; Bai et al. 2018). Furthermore, seminal exosomes can stimulate the secretion of prolactin by endometrial stromal cells during the window of implantation (WOI). This action enhances the in vitro decidualization process of human endometrial stromal cells (Rodriguez‐Caro et al. 2019). These findings suggest that seminal exosomes have a diverse range of mechanisms through which they can influence embryo implantation.

Extensive research indicates that prostasomes significantly impact the female reproductive tract's response to male antigens. Isolated human prostasomes can interact with lymphocytes, leading to the inhibition of their proliferation and phagocytosis (Skibinski et al. 1992). Exosomes derived from the sperm‐rich fraction of boar semen have been studied for their ability to induce changes in gene expression in endometrial epithelial cells in vitro. These exosomes were found to trigger the expression of transcripts such as CXCL2, CXCL8, CCL20, and TNFSF15, which are known to be upregulated in the endometrium following natural mating (Bai et al. 2018). This suggests that seminal exosomes have the capability to enhance the expression of genes related to immune and inflammatory responses in porcine endometrial tissue, mirroring the levels observed in the endometrium of naturally mated pigs.

Human prostasomes exhibit a variety of immune‐modulatory properties, including the natural killer (NK) cell activating receptor CD244 ligand, CD48 (Tarazona et al. 2011). When prostasomes are cultured with NK cells in vitro, they suppress CD244, leading to impaired NK cell degranulation and reduced expression of interferon‐gamma (IFN‐γ) in NK cells. Prostasomes contain CD59 and CD44, which play roles in inhibiting complement‐mediated lysis (Rooney et al. 1993; Babiker et al. 2005). CD59 plays a role in inhibiting the formation of the membrane attack complex (Rooney et al. 1993; Babiker et al. 2005), while CD46 functions as a cofactor for the proteolytic inactivation of complement factors C3b and C4b (Kitamura et al. 1995). Additional immune‐regulatory molecules linked to prostasomes include galectin‐3 (Jones et al. 2010) and its ligand, Mac‐2 binding protein (Block et al. 2011), along with the antibacterial human cationic antimicrobial protein‐18, which generates the antimicrobial peptide LL37 (Andersson et al. 2002). This antimicrobial activity may have implications for protecting against infections in the reproductive tract.

Proteomic and RNA sequencing analyses have revealed that epididymosomes transport cargo with the potential to modulate maternal immune function. This influence may manifest through the transfer of signaling factors onto sperm or through direct interactions within the female reproductive tract subsequent to ejaculation (Reilly et al. 2016). Human epididymosomes are known to transport various immune regulatory molecules, such as programmed cell death 6, galectin 3, and complement activators (Thimon et al. 2008; Théry et al. 2009). In mice, miRNAs acquired during transit through the epididymis possess the ability to regulate crucial pathways in the female immune response, including those related to TGF‐β signaling (Reilly et al. 2016; Nixon et al. 2015). These findings highlight the role of epididymosomes in modulating immune responses within the female reproductive system and suggest a mechanism by which these vesicles can impact maternal immune function.

The sncRNAs enclosed within exosomes present in semen could be transferred to recipient cells in the female reproductive tract, thereby influencing their gene expression patterns (Robertson and Sharkey 2016; Bai et al. 2018). This transfer of sncRNAs may play a role in regulating processes such as implantation and immune responses, which are essential for the success of a pregnancy (Robertson and Sharkey 2016). The ability of exosomal miRNAs to modulate gene expression in distant tissues has been demonstrated in various systems, suggesting a mechanism by which these exosomal miRNAs could potentially impact gene regulation in target cells within the female reproductive tract (Thomou et al. 2017; Castaño et al. 2018).

## 8 Seminal Exosomes in Programming Offspring Development (Postimplantation)

The compositions of semen influence not only fertility but also have lasting effects on offspring development. While seminal plasma is not obligatory for successful reproduction, evidenced by the effective use of ART with different sperm sources, it may still impact embryo development and the long‐term health of offspring (Robertson and Sharkey 2016; Watkins et al. 2018; Yin et al. 2022). Emerging evidence suggests that seminal plasma, along with its RNA components, especially RNAs originating from the epididymis, likely plays a crucial role in sperm function, fertilization, early embryogenesis, and potentially in shaping the phenotype of offspring (Jodar 2019). Paternal RNAs can be categorized based on their transcriptional source into three main groups: (1) RNAs transcribed in the testes and selectively retained within sperm, (2) RNAs originating externally to the testes, acquired by spermatozoa from exosomes released by the epididymis or other accessory sex glands during sperm maturation, (3) RNAs with an origin outside the testes, enclosed within exosomes present in semen, which directly interact with the female reproductive tract.

There is compelling evidence conducted in mice for the significant role of sperm‐borne RNAs in early embryonic development. The diminished developmental potential of embryos produced using sperm with disrupted sncRNA profiles, resulting from testicular conditional knockouts of *DICER* and *DROSHA*, was restored by injecting wild‐type sperm RNAs (Yuan et al. 2016). Subsequent investigations in mice revealed a decreased percentage of embryos progressing to the blastocyst stage and a reduced live birth rate when mature oocytes were injected with sperm containing 90% less RNA compared with controls (Guo et al. 2017). Interestingly, this developmental impairment was rectified by injecting wild‐type sperm RNAs into the embryos, indicating the crucial influence of sperm‐borne RNAs on embryogenesis (Guo et al. 2017).

Animal studies have demonstrated that the profile of sncRNAs within epididymosomes can be influenced by paternal environmental exposures, and these modifications are correspondingly observed in the sncRNA profile of sperm under comparable circumstances (Yin et al. 2022; Rompala et al. 2018; Chan et al. 2020). For instance, exposure of male mice to alcohol or the stress hormone corticosterone can induce alterations in the sncRNA content of both epididymosomes and sperm (Rompala et al. 2018). In a paternal stress model, co‐incubation of unexposed sperm with epididymosomes from exposed males before generating offspring through ICSI resulted in offspring displaying disrupted stress responses similar to those seen in offspring from naturally mated stressed fathers (Chan et al. 2020). These findings suggest a direct involvement of epididymosome cargo in influencing altered phenotypes in offspring, highlighting the potential impact of paternal exposures on the transmission of epigenetic information during sperm maturation and subsequent fertilization.

Variations in the abundance of specific sRNAs are observed between sperm collected from the caput and cauda epididymis in mice. While caput sperm display elevated levels of tRF‐Glu‐CTC and tRF‐Gly‐GCC, the levels of tRF‐Val‐CAC, a similarly prevalent sRNA, are significantly higher more than 10‐fold in cauda sperm compared with caput sperm (Sharma et al. 2016). Furthermore, there are notable fluctuations in the levels of certain miRNAs during sperm maturation in the epididymis (Sharma et al. 2018). These miRNAs are believed to be transferred to developing sperm through fusion with epididymosomes, illustrating a mechanism through which epididymal maturation influences the miRNA composition of sperm during their passage through the epididymis. For instance, miRNAs from certain genomic clusters, including the X‐linked miR‐880 cluster and the miR‐17‐92 oncomiR cluster, are significantly more abundant in cauda sperm compared with caput sperm in mice (Sharma et al. 2016; Nixon et al. 2015). When comparing embryos produced using testicular or cauda‐epididymal sperm with those generated from caput‐epididymal sperm in mice, it was observed that embryos derived from caput‐epididymal sperm exhibited decreased implantation rates and alterations in postimplantation development. Notably, the preimplantation molecular defects were fully rectified through the injection of sncRNAs derived from cauda epididymosomes (Conine et al. 2018). Furthermore, a comparison revealed that 100 miRNAs were expressed at lower levels in caput epididymal sperm than in germ cells retrieved from the cauda epididymis. This difference suggests a potential mechanism for the observed implantation defects in embryos generated from caput‐epididymal sperm, indicating differential regulation of miRNA expression as a potential contributing factor to these developmental irregularities (Conine et al. 2018).

The viability of a pregnancy is largely contingent upon the maternal immune system's tolerance toward the semiallogeneic fetus. Consequently, the immunological environment at the maternal‐fetal interface plays a pivotal role in initiating and sustaining pregnancy (Zhang et al. 2023). It is believed that decidual macrophages, constituting the second‐largest cellular population at the maternal‐fetal interface, play a crucial role in promoting the immune tolerance necessary for a successful pregnancy (Jiang and Li 2024). Seminal plasma facilitates the M2‐type polarization of decidual macrophages. Analysis through gene chip sequencing revealed elevated levels of miR‐26‐5p in seminal exosomes, which target the PTEN/PI3K/AKT pathway, effectively enhancing M2 polarization in decidual macrophages. This polarization creates an immune‐tolerant environment supportive of embryo implantation and development. Additionally, supplementation with seminal exosomes significantly reduced embryo resorption in spontaneously aborted mice, suggesting a potential therapeutic avenue for managing pregnancy complications (Zhang et al. 2025, 2024).

## 9 Seminal Exosomes and Pregnancy‐Associated Diseases (Recurrent Implantation Failure, Recurrent Pregnancy Loss, Pre‐Eclampsia)

Seminal fluid plays a pivotal role in facilitating successful embryo implantation and promoting optimal placental development (Shen et al. 2023; Schjenken et al. 2021; Morgan et al. 2020; George et al. 2020). Experiments have demonstrated that seminal exosomes interact with cells in the female reproductive tract. They actively participate in various processes, including modulating maternal immunologic response and metabolic adaptations essential for embryo implantation and establishment of a healthy pregnancy (Paktinat et al. 2019; Bai et al. 2018; D. Wang et al. 2021; Tarazona et al. 2011). Impaired immune adaptation during pregnancy can lead to complications such as recurrent implantation failure (RIF), recurrent pregnancy loss (RPL), and pre‐eclampsia (D. Li et al. 2021; Deer et al. 2023; Green and Arck 2020; Jena et al. 2023). Although exosomes are essential for mammalian pregnancy, alterations in their contents can contribute to the development of pregnancy‐related disorders (Motawi et al. 2018; Zhang, Li, et al. 2020; Nazri et al. 2020; Shen et al. 2022). Recent studies have highlighted that abnormal expression of proteins and noncoding RNAs within seminal exosomes may serve as potential factors in the onset of pregnancy‐associated diseases (Murdica, Cermisoni, et al. 2019; García‐Rodríguez et al. 2018; J. Sun et al. 2021).

Exposure of endometrial stromal fibroblasts to seminal exosomes results in increased secretion of prolactin and enhanced decidualization via interleukin‐11 signaling pathway to facilitate embryo implantation (Rodriguez‐Caro et al. 2019; George et al. 2020). The internalization of exosomes from seminal plasma by endometrial stromal cells and the subsequent release of IL‐6 and IL‐8 suggest their involvement in modulating the local inflammatory environment in the endometrium. This interaction between seminal plasma exosomes and endometrial stromal cells has implications for the success or failure of embryo implantation in ART (Paktinat et al. 2019; Pantos et al. 2022). The content of seminal exosomes from males with oligoasthenoteratospermia may contribute to impaired endometrial receptivity and potentially affect successful embryo implantation. Studies have reported that treatment of endometrial epithelial cells with seminal exosomes from males with oligoasthenoteratospermia resulted in reduced expression of endometrial implantation‐related genes, including MUC1, LIF, G‐CSF, CX3CL1, and VEGF. In comparison, seminal exosomes from individuals with normal sperm parameters did not elicit the same impact on gene expression (Gholipour et al. 2023).

Prospective randomized clinical trials have provided evidence that the utilization of seminal plasma can increase implantation rate and improve the clinical pregnancy rate in RIF patients (Coulam and Stern 1995; Wang and Lin 2021). Consistent with these findings, it has been indicated that seminal exosomes contain miR‐181a‐5p, which can enhance migration, invasion, and angiogenesis in the HTR‐8/SVneo trophoblast cell line through the suppression of PROX1 expression and subsequently Notch1 signaling activation. Interestingly, it has been observed that Notch1 is underexpressed in the villi tissue of women who conceived through in vitro fertilization (IVF) compared with those who conceived naturally through intercourse. This downregulation of Notch1 may contribute to early pregnancy loss in IVF cycles (Peng et al. 2021).

In investigations concerning RPL, studies have conducted proteome profiling and bioinformatics analyses of seminal exosomes. These analyses have revealed DEPs that are enriched in various biological pathways, encompassing “DNA replication, recombination and repair,” “cellular assembly and organization,” “gene expression,” “cell death,” and “survival pathways.” Additionally, the impact of these DEPs extends to disease pathways associated with “developmental, hereditary” and “immunological disorders” (Jena et al. 2021; Samanta et al. 2019).

In cases of unexplained RPL, the involvement of male factors has been highlighted as a potential contributing factor. Using differential proteomic analysis of seminal exosomal proteins, researchers explored potential paternal factors associated with RPL. Among the 447 identified DEPs, 385 were observed to be underexpressed and 62 overexpressed in individuals with RPL. Furthermore, 63 proteins were unique to the control group, while 59 proteins were specific to RPL cases. Pathway analysis utilizing tools such as Ingenuity Pathway Analysis revealed that developmental, hereditary, and immunological disorders were prominent among the top diseases associated with these DEPs. Functions related to cell death and survival, cellular assembly and organization, DNA replication, recombination and repair, as well as gene expression were notably disrupted in spermatozoa from individuals experiencing RPL. Specific proteins such as HNRNPC and HNRNPU, which were found to be overexpressed in RPL cases, may contribute to abnormalities in chromatin organization and telomere shortening. Conversely, the reduced expression of RUVBL1 could potentially lead to compromised centrosome function, affecting embryo development negatively (Samanta et al. 2019).

Pre‐eclampsia is a serious pregnancy complication and multisystem condition that can lead to severe high blood pressure and dysfunction or failure of the organs. The main cause of pre‐eclampsia is abnormal placentation, leading to deficient placental vascularization (Karrar and Hong 2023). The balance of pro‐inflammatory and immunomodulatory factors in seminal plasma plays a critical role in the process of placentation (Ahmadi et al. 2022; Redman and Sargent 2010). Evidence suggests that prolonged exposure to semen during pregnancy reduces the risk of pre‐eclampsia, and in contrast, short duration or prevention of seminal exposure increases the risk of developing pre‐eclampsia (Klonoff‐Cohen et al. 1989; Kho et al. 2009; Saftlas et al. 2014; Hendin et al. 2023). This indicates that the presence of seminal fluid has a protective effect against pre‐eclampsia. The exact mechanisms underlying this protective effect are not fully understood. However, it is believed that the pro‐inflammatory and immunomodulatory factors present in seminal plasma contribute to healthy placentation and proper placental vascularization, thereby reducing the risk of pre‐eclampsia.

Prostasomes contain E prostaglandins, which can induce the expression of prostaglandin‐endoperoxide synthase 2 (PGE2) and modulate prostaglandin E2 receptor levels in cervical cells (Oliw et al. 1993; Joseph et al. 2013). Increased concentrations of PGE2 can then act on EP4, a specific subtype of prostaglandin E2 receptor found on platelets, to suppress platelet aggregation (Philipose et al. 2010). Subsequently, circulating platelets are exposed to soluble microparticles in an inflamed vascular system and become activated. This activation is likely influenced by the interaction between PGE2, EP4 receptors, and the inflammatory environment. The activated platelets undergo degranulation and release various molecules that modulate their interactions with leukocytes and endothelial cells. Platelet‐leukocyte interactions lead to neutrophil infiltration into inflamed microvasculature, while platelet‐endothelial cell interactions result in inflammatory responses (Sahin et al. 2014). Accordingly, it is hypothesized that lower levels of prostaglandins in seminal plasma, including prostasomes, might disrupt the typical antiplatelet and anti‐inflammatory effects, leading to an increased risk of thrombotic events in the context of pre‐eclampsia (García‐Montalvo et al. 2016). This cascade of events underscores the intricate interplay between prostasomes, prostaglandins, platelet activation, and cellular interactions within the context of inflammation and vascular dynamics.

## 10 Role of Seminal Exosomes in Infections

Seminal infections pose a significant risk factor for male reproductive health, impacting various aspects such as spermatozoa development, sperm quality, and the potential for seminal tract obstruction (Lorusso et al. 2010; Teixeira et al. 2021; Castrillón‐Duque et al. 2018; Farsimadan and Motamedifar 2020). Besides, some bacterial, viral, and fungal infections can be transmitted during intercourse via semen and threaten the partner's health and fertility (Gimenes et al. 2014). Such infections can be transmitted to the embryo during fertilization, potentially disrupting embryonic development and trophoblast cell invasion, elevating the risk of miscarriage, and negatively impacting the health of the offspring (Farsimadan and Motamedifar 2020; Gimenes et al. 2014; Loutradi et al. 2006). An increasing body of research indicates that infections with pathogenic organisms cause substantial changes in exosome content (Lundy et al. 2015; Li et al. 2013; Gallo et al. 2017; Buck et al. 2014). Seminal exosomes exhibit bidirectional regulatory roles in pathogen infections. They either promote or prevent the infection process via transmission of pathogen‐ and host‐derived molecules (Vickram et al. 2021; Su et al. 2021). This bidirectional influence can lead to exosomes promoting infections through several ways: (Perry et al. 2013) transmission of pathogen‐related molecules (Rodriguez‐Martinez et al. 2021), facilitating pathogen immune evasion (McGraw et al. 2015), inhibiting immune responses by promoting apoptosis in immune cells. Conversely, exosomes also play a role in combating infections by: (Perry et al. 2013) directly inhibiting pathogen proliferation and (Rodriguez‐Martinez et al. 2021) stimulating immune responses involving monocytes, macrophages, NK cells, T cells, and B cells. Exosomes are thought to function as “bridges” during pathogen infections, facilitating various mechanisms and playing a crucial role in the biological processes that follow such infections. Alterations in exosome quantity, content, and membrane structure have been observed in this context (W. Zhang et al. 2018). Infections can lead to modifications in the structure of exosomal membranes, affecting the levels of structural proteins and lipids, and even causing spatial configuration changes. Moreover, the protein composition of exosomes can be altered under pathological conditions or during periods of stress (Conde‐Vancells et al. 2008).

Exosomes possess immunosuppressive properties, such as inhibitory effects on lymph‐proliferative responses, leukocytic ROS production, phagocytic activity and NK cell functions, to make immune tolerance toward sperm and semen antigens within the female body (Vojtech et al. 2014; Tarazona et al. 2011; Kaminski et al. 2019; Vojtech et al. 2016). Although immunosuppressive components of seminal exosomes maximize the chances of successful fertilization, they can also inadvertently promote the establishment of infections by creating an environment that enables infectious agents to evade the immune system (Kaminski et al. 2019). Moreover, it has been demonstrated that exosomes and microvesicles bind to and enter antigen‐presenting cells (APCs) in an APC–T cell co‐culture system. This interaction leads to a decrease in antigen‐specific cytokine production, degranulation, and cytotoxicity by antigen‐specific memory CD8+ T cells. Additionally, there is an increase in the expression of indoleamine 2,3‐deoxygenase, an enzyme that inhibits T‐cell function. Consequently, seminal exosomes can influence subsequent immune responses, which viral infections may exploit to weaken adaptive cellular immunity (Kaminski et al. 2019).

As an illustration, the primary source or vector for human immunodeficiency virus (HIV) infection is semen (Jewanraj et al. 2021). Individuals infected with HIV produce seminal plasma‐derived EVs, including exosomes, to induce pro‐inflammatory responses in endometrial epithelial cells and endometrial stromal fibroblast cells, potentially enhancing HIV transmission (Marques de Menezes et al. 2020). Seminal exosomes can suppress HIV infection and the production of pro‐inflammatory cytokines induced by HIV, independent of the activation status of primary lymphocytes. These exosomes modulate the expression of anti‐ and pro‐inflammatory regulators in a coordinated but asynchronous manner. They likely contain factors that are crucial in orchestrating processes that govern both HIV infection and the inflammatory responses triggered by HIV. In this context, seminal exosomes function as negative regulators of HIV replication in primary human lymphocytes. They selectively modulate the fundamental cellular programs necessary for HIV infection, encompassing both basal and activated states, thereby playing a crucial role in regulating HIV infection and associated inflammatory responses (Welch et al. 2020).

On the other hand, in infectious conditions, seminal exosomes can counteract their immunosuppressive properties through their antiretroviral and antibacterial activities. It has been shown that seminal exosomes from healthy individuals possess the ability to suppress the replication of retroviruses such as various strains of HIV‐1 in different cell types by inhibiting the activity of the intravirion reverse transcriptase enzyme (Madison et al. 2014). Treatment of vaginal epithelial cells with exosomes derived from healthy human semen blocked the transmission of HIV‐1 from vaginal cells to target cells in vitro. Moreover, in vivo study showed that human semen exosomes also inhibited intravaginal transmission and propagation of the murine acquired immunodeficiency syndrome (AIDS) virus in a mouse model (Madison et al. 2015). In the case of the Zika virus, known to cause Zika fever, which can also be transmitted via sexual intercourse and poses a significant risk of severe birth defects in pregnant women. There is evidence suggesting that seminal exosomes, derived from healthy donors, could restrict Zika virus binding to and infection of genital cells and tissues. This restriction mechanism occurs through the disruption of Zika virus membrane integrity, thereby preventing its successful infection (Wang et al. 2020).

Semen exhibits potent coagulating properties, leading to rapid healing of any abrasions and bleeding that may occur during intercourse. Seminal coagulant activity could potentially reduce the chance of transmitting blood‐borne infections like HIV. Prostasomes participate in this coagulant activity. Their membranes contain tissue factor (CD142) which is a vital clotting cofactor for factor VII (Fernandez et al. 1997). Prostasomes also engage in immunomodulation as well (Poliakov et al. 2009). One of their key functions is the regulation of complement activation through the presence of membrane co‐factor protein (CD46). Prostasomes have been shown to possess the ability to inhibit measles virus infection in Vero cells, a cell line commonly used in virology research. This suggests that they contribute to the immunomodulatory activity of human semen and may protect against certain viral infections, including measles virus, which is a cause of maternal and fetal morbidity and mortality (Kitamura et al. 1995).

Indeed, studies have indicated that prostasomes possess bacteriostatic properties as well. They contain various antimicrobial components, including cationic antimicrobial protein (hCAP‐18), chromogranin B, synaptophysin, and lipopolysaccharide‐binding protein (LBP). LBP, in particular, is an acute‐phase protein that plays a crucial role in the defense against gram‐negative bacteria. The presence of these antimicrobial components within prostasomes contributes to their ability to inhibit the growth and proliferation of bacteria (Andersson et al. 2002; Malm et al. 2005; Carlsson et al. 2003), underscoring their multifaceted role in the immune defense mechanisms present in human semen. It was found that galectin‐3 presented on the surface of prostasomes acts as an immunomodulator which could interact with bacterial lipopolysaccharides and antibacterial protein (FALL‐39) to participate in the antibacterial activity of semen (Jones et al. 2010; Kovak et al. 2013). Prostasomes have also demonstrated dose‐dependent growth inhibitory effects on *Bacillus megaterium* and several other bacterial strains. This further highlights the role of prostasomes in the innate immune defense of the male reproductive system, specifically in the defense against bacterial infections (Carlsson et al. 2003, 2000; Kumar 2019). Therefore, further research is necessary to fully comprehend the mechanisms underlying their antiviral and antibacterial activities and explore their potential applications in combating infectious diseases.

## 11 Application of Exosomes: Advantages and Challenges

### 11.1 Advantages of Exosomes as Therapeutic Agents (Delivery Vehicles, Immunomodulatory and Anti‐Inflammatory Properties, Stability, and Biodistribution)

Numerous contemporary medication candidates, including proteins and nucleic acids, exhibit notable instability in vivo, thereby presenting substantial obstacles to the achievement of efficacious therapeutic outcomes. In light of the challenges inherent in several existing nanoparticulate delivery methods, the utilization of exosomes as a surrogate for “delivery systems inspired by nature” presents an opportunity for the transportation of biological molecules (Ha et al. 2016; Basu and Ludlow 2016). Exosomes have garnered considerable attention due to their involvement in pathobiological mechanisms and are currently being explored as a potential tool for disease diagnosis and treatment (McGraw et al. 2015; Juyena and Stelletta 2012). In contrast to conventional nanoparticulate systems (polymeric nanoparticles or liposomes), exosomes possess the unique capability to circumvent the endosomal pathway and avoid lysosomal degradation, thereby facilitating cargo delivery into the cytoplasm (Mehrotra and Tripathi 2015). The enhancement of the transfection effectiveness for molecules like siRNA may be achieved by bypassing the endosomal pathway (del Pozo‐Acebo et al. 2021; Alvarez‐Erviti et al. 2011). Exosomes, with their inherent stability and precise targeting capabilities, offer significant advantages as drug delivery vehicles (L. Sun et al. 2021; J. Wang et al. 2021).

Due to their intrinsic tiny size and composition, exosomes possess the ability to evade macrophage phagocytosis and destruction, hence enabling prolonged circulation inside the human body. Numerous studies have demonstrated the lack of immune response induction in organisms receiving exosome therapy (Koh et al. 2020; Hu et al. 2020; Rezabakhsh et al. 2021; Saribas et al. 2020; Kharazi and Badalzadeh 2020). These vesicles possess the ability to influence target molecules in recipient cells, such as by dampening inflammatory responses, thereby establishing their potential as therapeutic modulators (Pillay et al. 2020; Williams et al. 2013). The complex nature of exosomes and their function in general health and disease conditions make it challenging to accurately predict their long‐term safety and therapeutic effects due to a lack of sufficient information (Dimik et al. 2023). A more profound comprehension of in vivo drug trafficking, the biological fate of exosomes, and their impact on targeted organs is necessary. In recent years, exosomal drug delivery and exosome‐based therapies have garnered significant attention, as exosomes serve as natural biological carriers with low immunogenicity and minimal toxic side effects (Shan et al. 2022; Ozkocak et al. 2022). The presence of exosomes has revolutionized the field of cell‐free treatments and has brought significant benefits. To utilize the potential of exosomes as therapeutics for various diseases and disorders, it is essential to evaluate their characteristics, including size, origin, systemic biodistribution, purification methods, and administration efficiency.

### 11.2 Seminal Exosomes in Reproductive Medicine

Seminal proteins play crucial roles in sperm development, sperm maturation, and fertilization processes. Meanwhile, seminal exosomes account for approximately 3% of the total proteins present in seminal plasma (Baskaran et al. 2020). Consequently, seminal exosomes and their protein components, such as ANXA2, play a critical role in determining sperm quality and fertilization potential (Sullivan et al. 2005). Recently, regenerative medicine techniques have gained prominence in addressing male infertility, particularly in cases of poor sperm quality, reflecting a growing emphasis on innovative solutions for enhancing reproductive outcomes in men (Tamadon et al. 2019; Bhartiya 2018; Sobhani et al. 2017). Current therapeutic approaches for male infertility encompass cell‐based methods like mesenchymal stem cell (MSC) therapy (Qian et al. 2020; Xing et al. 2016; Bahmyari et al. 2024) or cell‐free treatments such as exosome therapy (Guo et al. 2021; Phinney and Pittenger 2017; Liu et al. 2022; Şimşek et al. 2023). Exosomes, emerging as a novel therapeutic avenue in addressing male infertility‐related conditions, appear to offer solutions to the challenges encountered with MSC therapies in clinical settings. Recent advancements in exosome‐based strategies present a noninvasive and promising approach for rejuvenating spermatogenesis and promoting sperm regeneration, potentially revolutionizing treatments for male infertility‐related disorders (Vilanova‐Perez et al. 2020; Panda et al. 2021). The therapeutic potential of AF‐derived exosomes in a rat model of azoospermia demonstrated significant improvements in both spermatogenesis index and sperm parameters following the injection of AF‐derived exosomes (Mobarak et al. 2021). Moreover, one of the challenges in IVF clinics is the occurrence of sperm cryoinjuries during the freezing‐thawing process and the destruction of spermatozoa induced by cryo‐stress. Notably, research has identified the protective effects of exosomes against sperm cryoinjuries, highlighting the potential of exosomes in mitigating damage caused by cryo‐stress and enhancing the viability of sperm cells (Du et al. 2016; Mahdavinezhad et al. 2022).

### 11.3 Technical and Biological Challenges

#### 11.3.1 Extraction and Purification Difficulties

To ensure the production of high‐quality exosomes for medical applications, it is crucial to employ appropriate extraction techniques, optimize culture conditions, and develop innovative engineering transformation methods (Han et al. 2023). Despite the potential of exosomes for therapeutic cargo loading, challenges persist in understanding their optimal assembly for effective medication delivery (Tran et al. 2015). One drawback of using exosomes is their susceptibility to damage during isolation and purification processes (Rezabakhsh et al. 2021). Currently, there are several exosome extraction techniques available, each with distinct merits (Zhang, Bi, et al. 2020). Enhancing the yield and purity of exosomes can be achieved through modifications to standard methods such as ultrafiltration, microfluidic isolation, immunoprecipitation, ultracentrifugation, and precipitation (Rezabakhsh et al. 2021; Dimik et al. 2023; Kibria et al. 2018). However, these techniques may impact the physicochemical properties of exosomes. Targeting exosomes effectively for therapeutic purposes presents another significant challenge (Kibria et al. 2018). The method of isolating exosomes plays a critical role in determining their purity and quality (Yamashita et al. 2018). While ultracentrifugation and precipitation are commonly used techniques, they may not be suitable for therapeutic applications due to the alterations in composition and size observed in exosomes obtained through these methods (Yamashita et al. 2018; Witwer et al. 2013; Bobrie et al. 2012; Kowal et al. 2016). Exosomes acquired via various extraction techniques can exhibit heterogeneity, meaning that individual exosomes may contain varying quantities and types of bioactive molecules (Willms et al. 2016). There is still a lack of clarity regarding the distinctions between exosomes derived from various sources, and a comprehensive understanding of their functional variances has yet to be completely established (Li et al. 2023; Mohammadi et al. 2024). Standardized protocols for extracting therapeutically viable exosomes are currently lacking, underscoring the need for further refinement in this area. Furthermore, it is important to acknowledge that the field of engineering exosomes in this context is still in its exploratory stage.

#### 11.3.2 Storage and Stability Issues

Storing exosomes presents a notable challenge due to the susceptibility of exosomal cargo to degradation. At room temperature, exosomal cargo can degrade, while storage at 4°C can lead to a significant decrease in exosomal protein levels (Rezabakhsh et al. 2021). To preserve exosomes, they can be stored at −80°C in phosphate‐buffered saline, with the addition of trehalose serving as a protective measure against cryodamage (Bosch et al. 2016). Maintaining the sterility of exosomes is crucial to prevent contamination by viruses or bacteria (Rezabakhsh et al. 2021). The stability and lifespan of exosomes in bodily fluids represent critical considerations (Kibria et al. 2018). Ensuring an adequate supply of exosomes is a key challenge, necessitating the development of methods to extend their half‐life and local persistence for clinical applications (Yamashita et al. 2018). Studies suggest that combining exosomes with biomaterials may offer an optimal solution to address this challenge (M. Chen et al. 2022). Advancements in nanotechnologies and microfluidics have facilitated the integration of microfluidics into exosome isolation processes. Optimized and integrated microfluidic chips are emerging as promising tools for advancing future research (Han et al. 2020; Woo et al. 2017; J. Chen et al. 2022). These technological innovations are expected to drive progress in exosome research and applications moving forward.

#### 11.3.3 Standardization and Safety Concerns

To ensure optimal drug targeting using exosomes, it is crucial to prioritize nontoxicity and nonimmunogenicity. Additionally, these systems should be thoroughly evaluated for their safety in both in vitro and in vivo settings to avoid adding to the body's overall burden (Han et al. 2023; Petroušková et al. 2022). Therefore, further research and development are necessary to explore the potential of exosomes while ensuring their safety and efficacy. Tailoring the targeting capabilities of exosomes should also be developed to enhance their specificity and minimize off‐target effects. By addressing these critical aspects, exosomes can be effectively utilized in drug delivery systems while maintaining safety and efficacy. Currently, exosome purification technologies, including ultrafiltration, immunoaffinity, and ultracentrifugation, are associated with several drawbacks. These methods involve expensive instruments requiring large sample volumes, which can lead to potential protein contamination. Moreover, the multiple isolation steps involved often result in low isolation efficiency, sample loss, and compromised exosome recovery and purity. Exploring novel approaches to enhance the structure and effectiveness of exosomes presents a distinct and promising avenue for improving their utility in medicine. By investigating innovative strategies to address these limitations, significant advancements can be made in optimizing the isolation, purification, and utilization of exosomes for various therapeutic applications in the field of medicine.

## 12 Conclusion and Future Perspectives

In summary, the growing body of research highlights the pivotal role of seminal exosomes not only in male reproduction but also in influencing offspring health. The molecular profile of spermatozoa undergoes significant changes during epididymal transit, resulting in the production of mature sperm capable of fertilization and supporting early embryonic development to ensure the birth of healthy offspring. Moreover, the contents encapsulated within seminal exosomes have the potential to modulate gene expression in cells within the female genital tract, potentially influencing critical processes such as the female immune response and embryo implantation. The scope of exosomal research in human reproduction is extensive, holding promise for advancements in understanding and improving reproductive outcomes. However, numerous questions remain unanswered, highlighting the need for future research to elucidate and address the complexities surrounding exosome‐mediated mechanisms in human reproduction.

During ART, the absence of exposure of the female reproductive tract to the seminal fluid (as bypassed during artificial insemination) may contribute to pregnancies resulting from IVF/ICSI cycles. Advancing our understanding of the possible functions of exosomes may open up new avenues for treatments, not only enhancing natural conception but also improving positive health outcomes in ART. Considering the role of seminal exosomes, it is suggested that modifications to certain protocols, such as sperm preparation methods and culture media, may improve ART outcomes. Further research is essential to assess the feasibility of embryo culture exposed to exosomes or the utilization of exosome‐enriched media for embryo transfer, offering potential avenues for optimizing ART procedures and improving reproductive success rates.

Exosomes possess the ability to mitigate medication side effects by encapsulating therapeutic agents and precisely delivering them to targeted sites within the body. Furthermore, the utilization of exosomes derived from various sources, coupled with advancements in nanotechnology and targeted delivery systems, presents opportunities for enhancing the accuracy and efficiency of exosome‐based therapies. The future of male fertility research appears promising, with the potential for developing innovative methods to assess reproductive health and implement strategies to enhance fertility outcomes. Continued investigation into exosome research, supported by preclinical studies and rigorously designed clinical trials, is crucial for thoroughly evaluating the safety, efficacy, and long‐term effects of exosome‐based therapies. These advancements hold the potential to revolutionize the field of male fertility, offering innovative and minimally invasive treatments for those experiencing reproductive challenges.

## Author Contributions

Shayesteh Mehdinejadiani created the concept and design of the study. Nahid Azad, Zeinab Dehghan, Zahra Khosravizadeh, Fatemeh Saberi, Delsuz Rezaee, Tayyebeh Pilehchi, and Nasim Goudarzi participated in data collection and writing of the draft of the manuscript. Shayesteh Mehdinejadiani and Nahid Azad critically revised the manuscript. Zeinab Dehghan designed the images. Kobra Mehdinejadiani and Elnaz Salahi reviewed English language editing and revised the final version of the manuscript. All authors contributed to the article and approved the submitted version.

## Conflicts of Interest

The authors declare no conflicts of interest.

## Data Availability

Data sharing not applicable to this article as no data sets were generated or analyzed during the current study.
